# Reliability and validity of the Thai version of the Shoulder Pain and Disability Index (Thai SPADI)

**DOI:** 10.1186/s12955-015-0333-2

**Published:** 2015-09-04

**Authors:** Chanwit Phongamwong, Apijaree Choosakde

**Affiliations:** Department of Rehabilitation Medicine, Phramongkutklao Hospital and Phramongkutklao College of Medicine, 315 Ratchathewi, Bangkok, 10400 Thailand

## Abstract

**Background:**

The Shoulder Pain and Disability Index (SPADI) is a good clinical tool to evaluate patients with shoulder pain, but has not been adapted to Thai version. The objectives of this study were to translate the English version and culturally adapt the SPADI to Thai version and to evaluate the internal consistency and construct validity of the Thai SPADI among Thai participants having shoulder pain.

**Methods:**

Following the cross-cultural adaptation guidelines stated by the American Association of Orthopedic Surgeons (AAOS) Outcome Committee, the SPADI was translated to Thai version (Thai SPASI). Thai participants with shoulder pain completed the three questionnaires, i.e., the Thai SPADI, bodily pain subscale of the Thai Short Form 36 second version (Thai SF-36 V2) and the Thai version of disabilities of the arm, shoulder, and hand (Thai DASH). Internal consistency of the Thai SPADI was measured using Cronbach’s alpha coefficient. Convergent and divergent validity was used to measure construct validity of the Thai SPADI by assessing the correlation of the Thai SPADI with the Thai DASH and bodily pain subscale of the Thai SF-36 V2.

**Results:**

Of 44 participants, the majority of participants were female (68.2 %) and had Bachelor’s degree or higher education level (59.1 %) with a mean age of 50.4 years (SD 14.3). Cronbach’s alpha coefficient of the Thai SPADI in pain subscale, disability subscale and total scale was 0.92, 0.94 and 0.95, respectively. The correlation of the Thai SPADI with the Thai DASH and bodily pain subscale of the Thai SF-36 V was 0.79 (p < 0.001) and -0.49 (p = 001), respectively.

**Conclusions:**

The Thai SPADI has excellent internal consistency and moderate to high construct validity to assess shoulder disability among Thais.

## Background

Shoulder pain is a common problem of musculoskeletal pain among the general population [1, 2]. The prevalence can rise to 72.2 % in specific populations such as dental personnel [3]. It could produce disability or disturb activity of daily living and sleep quality [4, 5]. The shoulder disability questionnaire is a useful tool for clinicians to evaluate and follow up patient’s symptoms after treatment. Many shoulder disability questionnaires are available such as the Disability of the Arm, Shoulder, and Hand scale (DASH), the American Shoulder and Elbow Surgeon Standardized Assessment Form (ASES) and the Shoulder Pain and Disability Index (SPADI).

The DASH is the most acceptable tool to evaluate patients with upper extremities disorders comprising a 30-item self-administrated questionnaire. It evaluates the disability to perform activity of daily living. In 2014, The DASH was translated to Thai (DASH-TH) with cross-cultural adaptation, and 40 patients with upper extremities problems were recruited to assess the validity and internal consistency. The results revealed good clinimetric quality of the Thai version [6]. Currently, only the DASH-TH is available to assess the disability related with shoulder pain among Thais but is not quite practical in clinical use because it requires a long time to complete and is not specific to shoulder disorders [7].

Hence, some practical and shoulder-specific disability questionnaires could be available in Thai. The SPADI, developed by Roach KE et al. in 1991, is the English self-reported questionnaire consisting of only 13 items divided in two subscales: pain and disability [8], and its questions are easy to understand, requires only a short time to answer, and is recommended for clinical assessment and research settings [7]. In addition, it was translated to many languages including Persian, Tamil, Danish, German, Slovenian, Italian and Portuguese [9–14]. For these reasons, the purposes of the present study were to translate English version and culturally adapt the SPADI to Thai version, and to assess its internal consistency and construct validity. The author hypothesized that the Thai SPADI would be a valid and reliable questionnaire to assess shoulder disability among Thais having shoulder pain.

## Methods

### Translation and cross-cultural adaptation

Because the SPADI was developed in English-speaking countries, its items must not only be translated well lexically but also must be adapted to Thai culture to maintain the content validity of the original version [15, 16]. Now, no consensus cross-cultural methods are available but most of them include use of committees, focus groups, and back translations [17]. In the present study, with the official permission by Kathryn E. Rouch, the Thai SPADI was translated by following cross-cultural adaptation guidelines stated by the American Association of Orthopedic Surgeons (AAOS) Outcome Committee. According to AAOS guidelines, the first step was completed by translating from English to Thai by two Thai native translators, i.e., one physician and one university lecturer in English literature. Next, this pre-final Thai SPADI was back translated to English by two English-native translators to confirm that the meaning and concept of the original version still remained. Then an expert committee including four translators and one rehabilitation physician who completed Master’s degree in clinical epidemiology discussed and revised the pre-final version. Finally, the pre-final version was used and tested to determine understanding of items at the Rehabilitation Department, Phramongkutklao Hospital [15].

### Testing internal consistency and construct validity

#### Participants

A convenient sampling was performed among patients with shoulder pain at the Rehabilitation Department, Phramongkutklao Hospital. Eligibility criteria included adults (18 years old or over) who natively communicate and were able to read and write in Thai. Those with cognitive, communication, or psychological problems were excluded. Fifty-two patients with musculoskeletal pain were willing to participate in this study. All were informed about study details then signed the informed consent document. However, eight participants were excluded from the evaluation due to incomplete questionnaires. The study was approved by the Institutional Review Board, the Royal Thai Army, Medical Department.

#### Questionnaires

Participants completed self-administered paper questionnaires consisting four parts including demographic characteristics, the Thai SPADI, the Thai DASH and the bodily pain subscale of Thai SF-36v2.

#### The SPADI

The SPADI is a self-reported questionnaire consisting of 13 items divided in two parts: pain and disability subscale. The pain subscale includes five questions about pain intensity at its worst and when lying on the involved side, reaching for something on a high shelf, touching the back of the neck and pushing with the involved arm. The disability subscale includes eight questions about difficulty when washing the hair, washing the back, putting on an undershirt or jumper, putting on a shirt that buttons down the front, putting on your pants, placing an object on a high shelf, carrying an object of 10 pounds (4.5 kilograms) and removing something from your back pocket. Each question of both pain and disability subscale was scaled in 11-numeric ratings ranging from 0 to 10. Each score was summed and transformed to percentage. Finally, the average score between pain and disability subscale comprised the total SPADI scores ranging from 0 (the best) to 100 (the worst) [8].

#### The DASH

The DASH is a self-reported 30-item questionnaire developed by the Institute for Work and Health (IWH) together with the AAOS to assess the disability of daily activity regarding arm, shoulder, and hand pathology. Nowadays, the DASH has been translated to more than 40 languages including Thai. The DASH scores range from 0 (the best) to 100 (the worst) [6].

### The bodily pain of the second version of short form-36 health survey (SF-36v2)

The SF-36v2 is a self-reported, health-related quality of life (HRQoL) questionnaire consisting of eight subscales: physical functioning, role limitations due to physical problems, bodily pain, general health, vitality, social functioning, role limitations due to emotional problems, and mental health. The questionnaire refers to the HRQoL in the past four weeks. The participants in the present study completed only two items of the bodily pain subscale including intensity of bodily pain and extent the pain interfered with normal work. Scores of the bodily pain subscale ranged from 0 (the worst) to 100 (the best). The Thai SF-36v2 showed acceptable reliability (The Cronbach’s alpha coefficient was 0.86) [18].

### Statistical analysis

Demographic characteristics were presented in mean with standard deviation and scored by percentage. Internal consistency of the Thai SPADI was measured using Cronbach’s alpha coefficient. Pearson’s correlation was performed to determine the correlation of the Thai SPADI with the Thai DASH (convergent validity), and with bodily pain subscale of the Thai SF-36v2 (divergent validity) for assessing construct validity. The strength of the correlation was determined according to Dancey and Reidy’s categorization [19]. All data were analyzed using STATA software (Stata Corp, College Station, TX, USA).

## Results

### Participants

Of 44 participants, the majority of participants were females (68.2 %) and had Bachelor’s degree or higher education level (59.1 %) with a mean age of 50.4 years (SD14.3). The mean score of Thai SPADI was 46.3 (SD 22.1) with a minimum score of 4.6 and maximum score of 93.1 (Table 1).

**Table 1:** Demographic data of the participants having shoulder pain and their Thai SPADI score

Characteristics	
Age (year) – Mean (S.D.)	50.4 (14.3)
Female – n (%)	30 (68.2)
Educational level – n (%)	
Primary school	4 (9.1)
Secondary school	12 (27.3)
Diploma	2 (4.5)
University	26 (59.1)
Thai SPADI score – Mean (S.D.)	
Pain subscale	56.4 (22.4)
Disability subscale	40.0 (24.4)
Total scale	46.3 (22.1)

### Internal consistency

The internal consistency of the Thai SPADI pain subscale, disability subscale and total scale was excellent. Cronbach‘s alpha coefficients were 0.92, 0.94 and 0.95, respectively (Table 2).

**Table 2:** Internal consistency of the Thai SPADI

	Cronbach’s alpha
Pain subscale (5 items)	0.92
Disability subscale (8 items)	0.94
Total scale (13 items)	0.95

### Construct validity

The Thai SPADI and Thai DASH showed a strongly positive correlation (r = 0.79, p <0.001) and moderately negative correlation between the Thai SPADI and bodily pain subscale of the Thai SF-36v2 (r = 0.49, p = 0.001) (Table 3).

**Table 3:** Construct validity of the Thai SPADI

	DASH	Bodily pain of SF36v2
Pain subscale	0.59	- 0.55
	*P* < 0.001	*P* < 0.001
Disability subscale	0.83	- 0.41
	*P* < 0.001	*P* = 0.005
Total scale	0.79	- 0.49
	*P* < 0.001	*P* = 0.001

## Discussion

The present study showed that the Thai SPADI demonstrated extremely good validity and reliability. The internal consistency in pain subscale, disability subscale and total scale were excellent, similar to the original version (English) of Roach KE et al.: Cronbach’s alpha = 0.95 [8] and other versions included Tamil; Cronbach’s alpha = 0.93 [12], German; Cronbach’s alpha = 0.95 [9] and Danish; Cronbach’s alpha = 0.94. The level of correlation of the Thai DASH with pain subscale, disability subscale and total scale of the Thai SPADI was moderate to strong, slightly lower than the English version (r = 0.76 for pain subscale and r = 0.83 for disability subscale) [20] and the German version (r = 0.76 for pain subscale, r = 0.89 for disability subscale, r = 0.88 for total scale) [9]. In addition, the bodily pain subscale of the Thai SF-36v2 showed a moderate correlation with Thai SPADI, also slightly lower than those of the English (r = 0.64) [21] and the German versions (r = 0.61) [9].

Our study had some limitations because fewer participants were enrolled compared with the study of Beaton DE et al. (n = 138 and 90) [20, 21] and Angst F et al. (n = 125) [9]. Additionally, enrolled participants were from the Rehabilitation Department of one tertiary hospital and might not represent the general Thai population. Most participants (59.1 %) had a good level of education making the results of the clinimetric properties of the present study reliable. However, the SPADI Thai version was rather simple and understandable to anyone at any level of education. This was supported by the study of Jeldi AJ et al. [11] showing excellent internal consistency among enrolled participants with an education at high school level or lower.

## Conclusions

The Thai SPADI is a self-reported shoulder disability questionnaire showing excellent internal consistency and moderate to high construct validity. Our study demonstrated that the Thai SPADI could be a useful questionnaires for Thai patients suffering from shoulder pain both in clinical practice and the research settings.

## Abbreviations

DASH: Disability of the Arm, Shoulder, and Hand scale
ASES: American Shoulder and Elbow Surgeon Standardized Assessment Form
SPADI: Shoulder Pain and Disability Index
SF-36v2: Second version of Short-Form 36 Health Survey
HRQoL: Health-Related Quality of Life
